# Physiology of renal glucose handling via SGLT1, SGLT2 and GLUT2

**DOI:** 10.1007/s00125-018-4656-5

**Published:** 2018-08-22

**Authors:** Chiara Ghezzi, Donald D. F. Loo, Ernest M. Wright

**Affiliations:** Department of Physiology, Geffen School of Medicine at UCLA, Los Angeles, CA 90095-1751 USA

**Keywords:** Gliflozins, Glucose, GLUTs, Inhibitors, Kidney, Phlorizin, Proximal tubule, Review, SGLTs, Type 2 diabetes mellitus

## Abstract

The concentration of glucose in plasma is held within narrow limits (4–10 mmol/l), primarily to ensure fuel supply to the brain. Kidneys play a role in glucose homeostasis in the body by ensuring that glucose is not lost in the urine. Three membrane proteins are responsible for glucose reabsorption from the glomerular filtrate in the proximal tubule: sodium−glucose cotransporters SGLT1 and SGLT2, in the apical membrane, and GLUT2, a uniporter in the basolateral membrane. ‘Knockout’ of these transporters in mice and men results in the excretion of filtered glucose in the urine. In humans, intravenous injection of the plant glucoside phlorizin also results in excretion of the full filtered glucose load. This outcome and the finding that, in an animal model, phlorizin reversed the symptoms of diabetes, has stimulated the development and successful introduction of SGLT2 inhibitors, gliflozins, in the treatment of type 2 diabetes mellitus. Here we summarise the current state of our knowledge about the physiology of renal glucose handling and provide background to the development of SGLT2 inhibitors for type 2 diabetes treatment.

**Electronic supplementary material:**

The online version of this article (10.1007/s00125-018-4656-5) contains a slideset of the figures for download, which is available to authorised users.

## Introduction

**Fig. 1:**
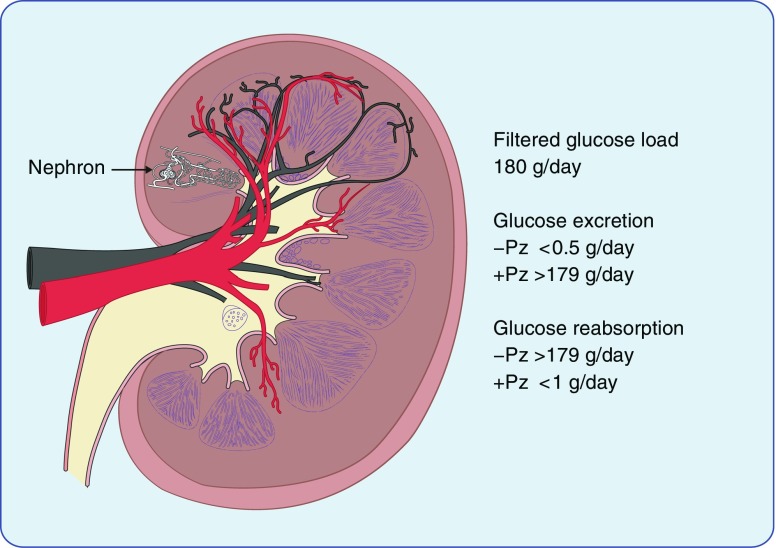
A drawing illustrating a cross-sectional view of the human kidney, showing the location of one of the 1 million nephrons in the kidney. The renal vein and renal artery are shown in black and red, respectively. The average daily filtered glucose load, urinary glucose excretion and glucose reabsorption in healthy adults are also shown. Phlorizin (Pz) increases glucose excretion to the filtered load and eliminates glucose reabsorption. This figure is available as part of a downloadable slideset

Although the kidneys freely filter plasma glucose, none appears in the urine. However, in diabetes, glucose may appear in the urine, specifically when the plasma glucose concentration is so high that the filtered load exceeds the maximum capacity for sugar reabsorption. Homer Smith and his colleagues were the first to quantify glomerular filtration rates in humans. They showed that all the filtered glucose was normally reabsorbed and, importantly, they also demonstrated that reabsorption of total filtered glucose load could be inhibited by a compound called phlorizin (Fig. 1) [1]. This prompted decades of research into the location and mechanism of kidney glucose transport, mostly in animal models.

### A brief history of the discovery of renal glucose transporters

**Fig. 2:**
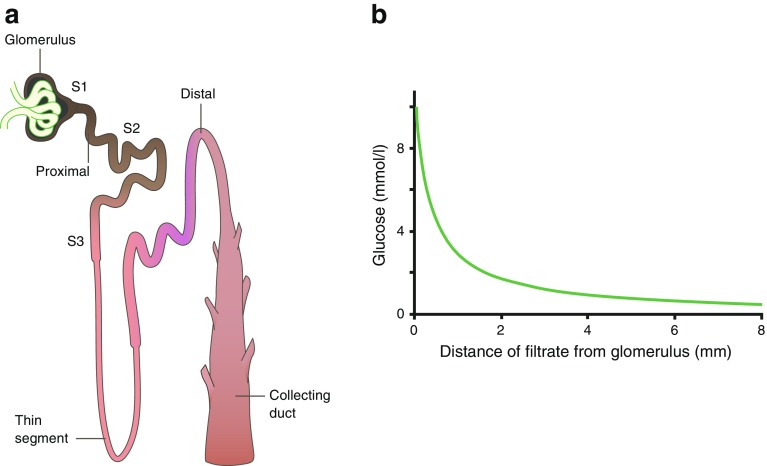
Glucose reabsorption by the proximal tubule. (**a**) Schematic representation of a single nephron, the functional unit of the kidney. (**b**) Glucose concentration (mmol/l) measured in micropuncture studies as fluid flows from the glomerulus along the tubule [51]. SGLT2 and GLUT2 are responsible for glucose reabsorption in the S1 and S2 segments, and SGLT1 and GLUT2 are responsible for glucose reabsorption in the S3 segment . Adapted from [6], distributed under the terms of the CC BY 4.0 Attribution License (http://creativecommons.org/licenses/by/4.0/). This figure is available as part of a downloadable slideset

Early micropuncture experiments on amphibians and rats found that glucose was completely reabsorbed in the proximal tubule (Fig. 2). Following the pioneering work of Mo Burg who introduced techniques for research on isolated perfused kidney tubules (reviewed in [2]), Barfuss and Schafer [3] found that the capacity for glucose absorption was tenfold larger in early (S2) rabbit proximal tubules than late (S3) tubules. Furthermore, they found that the affinity for glucose was higher in S3 tubules than S2 tubules, and that luminal phlorizin blocked absorption in both segments. This led to the concept that the bulk of glucose reabsorption occurred in the early proximal tubule (S1 and S2 segments), and that the later segment (S3) ‘mopped up’ the rest.

Our understanding of the mechanism of sugar transport has largely rested on the progress stemming from Bob Crane’s 1961 sodium–glucose cotransport hypothesis that glucose transport across the intestinal brush border membrane is uphill and coupled with the downhill movement of sodium. Stan Schultz and Pete Curran largely confirmed and expanded Crane’s hypothesis in the broader context of trans-epithelial transport [4]. Ultimate experimental proof of cotransport came from seminal work by Ulrich Hopfer and colleagues on isolated brush border membrane vesicles [5]. For a summary of the early history of the cotransport hypothesis, see [6]. The second step of intestinal glucose absorption, passive exit from the cell across the basolateral membrane, was thought to occur by facilitated diffusion. This two-stepped process explains how glucose absorption can occur against a concentration gradient across the intestinal epithelium (see [4]).

An early analysis of two rare genetic disorders of intestinal and renal glucose transport, familial renal glucosuria and glucose–galactose malabsorption, led to the hypothesis that two genes are primarily involved in glucose reabsorption in the kidney, with one of these genes also being expressed in the small intestine (giving rise to glucose–galactose malabsorption). Fast-forward to the present day and we now know that these genes encode the brush border sodium−glucose cotransporters (SGLT1 and SGLT2). Moreover, studies of Fanconi–Bickel syndrome, a genetic disease resulting in severe glucose loss in the urine [7], point to GLUT2 as the kidney basolateral glucose transporter [8, 9]. These genetic conditions, discussed later, have been instrumental in revealing the key players in renal glucose transport: SGLT1, SGLT2 and GLUT2.

In this review, we summarise the physiology of renal SGLTs and GLUTs, and provide rationale for the development of SGLT2 inhibitors to treat type 2 diabetes, as well as outlining the limitations of this therapeutic approach [10, 11].

## Cellular mechanisms for glucose reabsorption

**Fig. 3:**
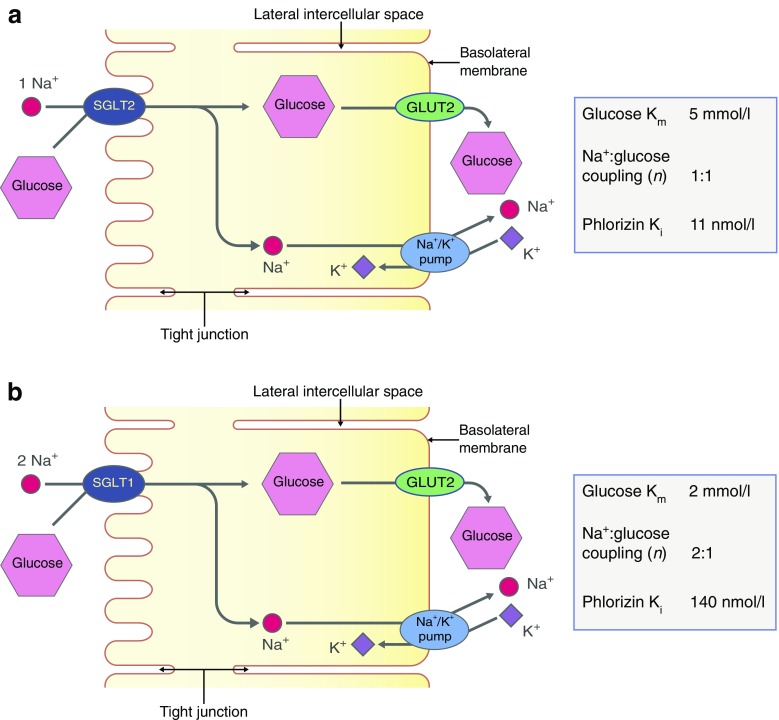
Reabsorption of glucose in the proximal tubule. (**a**) Epithelial cells of the S1 and S2 segments of the proximal tubule express SGLT2 on the apical membrane and GLUT2 on the basolateral membrane. (**b**) Epithelial cells of the S3 segment express SGLT1 on the apical membrane and GLUT2 on the basolateral membrane. In both S1/S2 and S3 segments, glucose reabsorption occurs, first via glucose transport across the apical membrane by SGLTs and then by passive glucose exit towards the plasma via GLUT2. The sodium gradient across the apical membrane is maintained by the basolateral Na^+^/K^+^ pump. At an extracellular NaCl concentration of 150 mmol/l, a membrane potential of −50 mV and at 37°C, the human SGLT2 has a K_m_ for glucose of 5 mmol/l, a K_i_ for phlorizin of 11 nmol/l and a sodium:glucose coupling ratio of 1:1. Under the same conditions, human SGLT1 has a glucose K_m_ of 2 mmol/l, a phlorizin K_i_ of 140 nmol/l, and a sodium:glucose coupling ratio of 2:1. Adapted from [6], distributed under the terms of the CC BY 4.0 Attribution License (http://creativecommons.org/licenses/by/4.0/). This figure is available as part of a downloadable slideset

The current models for glucose reabsorption from the glomerular filtrate by SGLT2 and SGLT1 in the different segments of the proximal tubule, S1/S2 and S3, are shown in Fig. 3. In both S1/S2 and S3 segments, the first stage is glucose transport across the apical membrane by SGLTs. This leads to glucose accumulation within the epithelium, modulated to some extent by intracellular metabolism. The glucose concentration gradient between the cell and plasma in turn drives the second stage: net passive exit of glucose through the basolateral membrane, towards the plasma, via GLUT2. The basolateral Na^+^/K^+^ pump (which extrudes *three sodium* ions for every two *potassium* ions entering the cell) maintains the sodium gradient across the apical membrane by pumping sodium out of the cell, towards plasma. Inhibition of the Na^+^/K^+^ pump by cardiac glycosides blocks the pumping of sodium out of the cell, with the concomitant rise in intracellular sodium concentration. The elimination of the sodium gradient across the apical membrane results in the loss of sodium–glucose cotransport across the apical membrane. Thus, the two-stage process, together with the absorption of glomerular fluid, accounts for the complete absorption of glucose by the time the filtrate reaches the end of the proximal tubule (Fig. 2).

## Cloning renal glucose transporters

In 1987, members of the Wright laboratory began pioneering work that resulted in the identification of SGLTs and their functional properties. The rabbit intestinal transporter was first identified by expression cloning [12], followed by homology cloning of the human intestinal SGLT1 and renal SGLT2 transporters [13, 14]. The SGLTs belong to a human gene family, *SLC5*, which contains 12 members, including sodium-coupled transporters for myoinositol, fructose, iodide and short-chain fatty acids [6, 15, 16]. *SGLT*2 (also known as *SLC5A2*) mRNA is almost exclusively expressed in the kidney, while *SGLT1* (*SLC5A1*) mRNA is found mainly in the small intestine and is only expressed to a small extent in the kidney [6, 17]. A single-cell transcriptomic study of mouse kidney has revealed that *Sglt2* is a unique marker gene for cells of the S1 segment of the proximal tubule [18]. *Glut2* (also known as *Slc2a2*) is expressed in both S1 and S3, while *Sglt1* is expressed at a low level along the proximal tubule, with a somewhat higher level in S3. The *SGLT* genes code for membrane proteins with 14 transmembrane helices, as confirmed by the crystal structures of a bacterial homologue, vSGLT [19, 20]. The crystal structures have also provided important clues about the SGLT transport mechanism (detailed below).

### Location of SGLTs in the kidney

The localisation of SGLT1 and SGLT2 in the kidney has been determined by immunohistochemistry using antibodies to the cloned transporters [21–25]. SGLT2 is found in the apical membrane of the S1 and S2 segments of the proximal tubule, while SGLT1 is restricted to the apical membrane of the S3 segment. In rodents, SGLT1 is also located in the apical membrane of the ascending limb of the loop of Henle, but the functional significance of this finding is unknown. We note that currently used immunocytochemical methods do not provide quantitative information about the density or functional activity of targeted membrane proteins. Actually, it is the number of SGLT proteins and their turnover number that determine the functional activity of SGLTs in the cell membrane. This information is not available for SGLT2 and SGLT1 in the apical membrane of S1/S2 and S3 segments.

### Functional properties

The functional properties of SGLT1 and SGLT2 have been determined by their expression in heterologous expression systems such as *Escherichia coli*, *Xenopus laevis* oocytes, and cultured cells lacking endogenous activity, e.g. human embryonic kidney cells (HEK 293) and African green monkey kidney, SV40 transformed cells (COS-7) (see [6, 16, 26]). In these systems, the kinetics of sodium–glucose cotransport have been determined as a function of extracellular and intracellular sodium, sugar and phlorizin concentrations and membrane potential. For now, it suffices to summarise that, at an extracellular NaCl concentration of 150 mmol/l, a membrane potential of −50 mV and at 37°C, the human SGLT2 has an apparent affinity constant (K_m_) of 5 mmol/l (K_m_ = the substrate concentration at which the transport velocity is one-half its maximal value), and a sodium:glucose coupling ratio of 1:1. In contrast, under the same conditions, human SGLT1 has a glucose K_m_ of 2 mmol/l and a sodium:glucose coupling ratio of 2:1 (Fig. 3). These properties are consistent with the hypothesis that, in humans, the bulk of glucose is absorbed in S1/S2 by SGLT2 and GLUT2, with complete reabsorption occurring in S3, enabled by the higher affinity of human SGLT1 for glucose and the 2:1 sodium:glucose coupling ratio.

### How do we know that the current models for glucose absorption in the proximal tubules are correct?

**Fig. 4:**
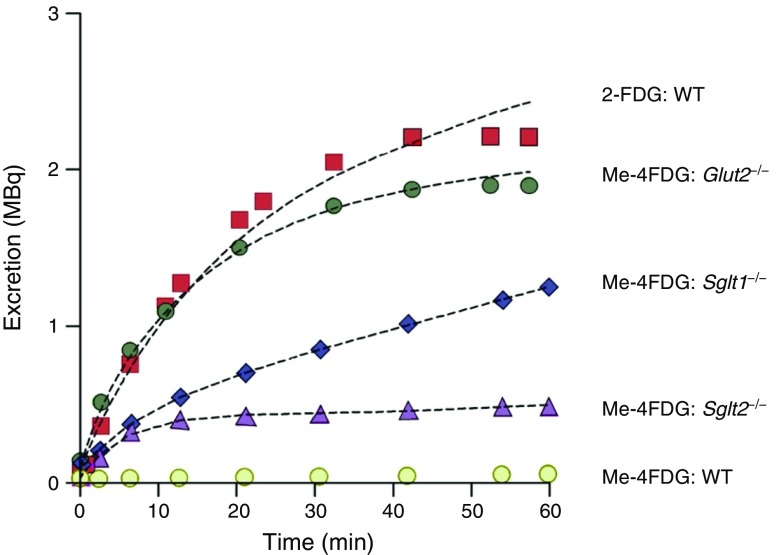
Urinary excretion of glucose PET tracers, 2-FDG and Me-4FDG in wild-type, *Glut2*^*−*/−^, *Sglt1*^−*/*−^ and *Sglt2*^−*/*−^ mice. The total amount of 2-FDG and Me-4FDG in the urinary bladder of representative mice as a function of time after intravenous injection of radiotracer (11 MBq) is shown. The data were fitted to a three-compartmental model for glomerular filtration, reabsorption and urinary excretion, showing that the excretion of 2-FDG in wild-type mice, and Me-4FDG in the Glut2^−/−^ mice was equivalent to the filtered glucose load. The excretion of Me-4FDG was greater in *Sglt1*^−*/*−^ than *Sglt2*^−*/*−^ mice. The entire filtered load of Me-4FDG was reabsorbed in wild-type mice. Adapted from [27] distributed under the terms of the CC BY-NC 4.0 Attribution License (https://creativecommons.org/licenses/by-nc/4.0/). This figure is available as part of a downloadable slideset

Evidence for the importance of SGLT1, SGLT2 and GLUT2 has been obtained using knockout mice and a non-invasive imaging method to monitor urinary excretion, micro positron emission tomography (microPET) [27]. Figure 4 shows the time course of excretion of two PET tracers into the urinary bladder of mice: 2-deoxy-2-fluoro-[^18^F]-d-glucose (2-FDG), which is a substrate for GLUT2 but not for SGLTs, and methyl-4-fluoro-[^18^F]-4-deoxy-d-glucopyranoside (Me-4FDG), a substrate for SGLTs with only low affinity for GLUT2. After intravenous injection of 2-FDG, the tracer is rapidly excreted into the urinary bladder of wild-type mice at a rate approximating the filtered load, consistent with the fact that 2-FDG is not a substrate for apical SGLTs. On the other hand, there is no measurable excretion of Me-4FDG, consistent with this tracer being a substrate for SGLTs in the apical membrane and a low-affinity substrate of GLUT2 in the basolateral membrane. In GLUT2 knockout mice, Me-4FDG is excreted at a rate comparable to 2-FDG. While, in SGLT1 and SGLT2 knockout mice, Me-4FDG is excreted, albeit less than the filtered load. Overall, these results are consistent with the roles of SGLT1, SGLT2 and GLUT2 in renal glucose reabsorption.

Further evidence comes from measurement of 24 h glucose excretion in SGLT1, SGLT2 and dual SGLT1/SGLT2 knockout mice [28]. In double knockout mice, the entire filtered glucose load is excreted in the urine, while in single SGLT2 and SGLT1 knockout mice, 67% and 98% of the filtered glucose load is reabsorbed, respectively. Some patients with truncation mutations in *SGLT2* have been found to excrete less than 50% of the filtered glucose load, while those with truncation mutations in *SGLT1* have only mild glucosuria (see: [29]; Table 7.1 in [30]; Table 6 in [6]). Finally, in both mice and humans the ‘knockout’ of functional GLUT2 (truncation mutations in *GLUT2* results in massive glucosuria [9].

Collectively, this data suggests that, in both mice and men, in the early proximal tubule (S1/S2), SGLT2 reabsorbs the bulk of the filtered glucose load and that, in the late proximal tubule (S3), SGLT1 provides a reserve capacity for up to 70% of the filtered load. In all three segments (S1, S2 and S3), GLUT2 in the basolateral membrane is essential for completing glucose absorption across the tubule.

## Inhibition of glucose reabsorption

External phlorizin acts as a high-affinity, specific, non-transported, competitive, dead-end inhibitor of sodium:glucose transport in and out of a cell. It contains a glucose structure on one end, which binds to the glucose-binding site on SGLTs, and an aglycone tail (phloretin), which binds to the wall of the hydrophilic cavity leading to the glucose-binding site (described further below). Internal phlorizin, even at high cytoplasmic sodium concentrations, is a poor inhibitor of both forward and reverse glucose transport [31].

**Fig. 5:**
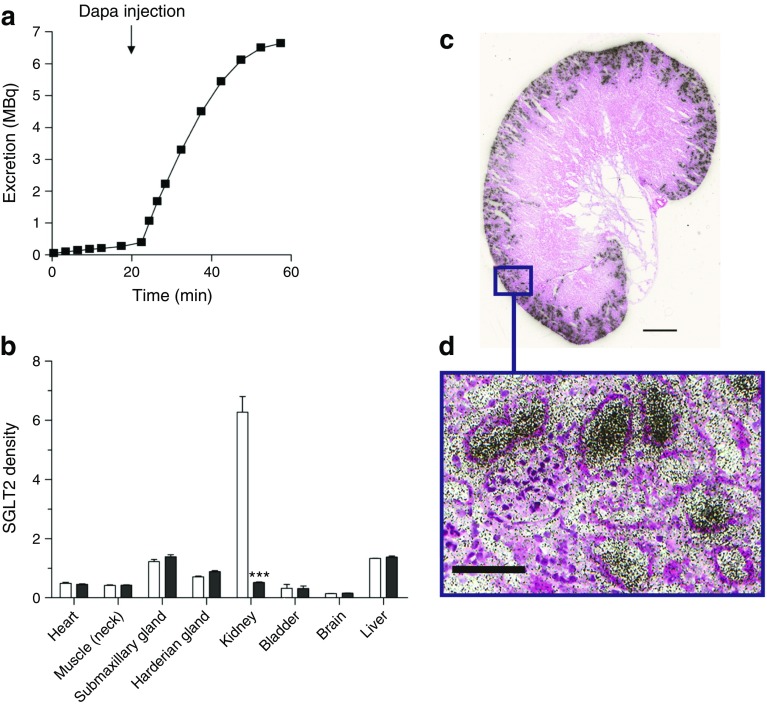
(**a**) Time course of Me-4FDG excretion into the urinary bladder of a rat. Me-4FDG (300 MBq) was injected intravenously into rats and excretion into the urinary bladder measured using microPET. Its excretion into the urinary bladder (MBq) is plotted as a function of time before and after injection of 1 mg/kg dapagliflozin (Dapa; an SGLT2 inhibitor) at 20 min. Injection of dapagliflozin causes the rapid excretion of Me-4FDG into the urinary bladder. Similar results were obtained with intravenous phlorizin (A. S. Yu and C. Ghezzi, unpublished results). (**b**) SGLT2 distribution in a rat, analysed using F-Dapa microPET. Animals were injected with F-Dapa (300 MBq) and the distribution of the tracer was imaged at 60 min using microPET. F-Dapa binding to organs is shown in control conditions (white bars) or after competition with cold dapagliflozin (black bars). Three-dimensional regions of interest (ROIs) were drawn over each organ and the data are presented as a percentage of the initial dose per tissue weight (%ID/g), with the exception of the bladder, for which values are presented as percentage ID per total bladder volume. Data are presented as the apparent density of SGLT2 in each organ, as means + SEM. ***p ≤ 0.001 vs control. The data show that functional SGLT2 is only expressed in the kidney. (**c**, **d**) Location of SGLT2 in the mouse kidney, visualised using F-Dapa and ex vivo microautoradiography and H&E staining. A mouse was injected with 148 MBq F-Dapa and after 15 min the kidney was removed and processed. (**c**) Aligned autoradiogram and H&E-stained image of the whole mouse kidney. Scale bar, 1 mm. (**d**) F-Dapa binding to the tubules surrounding a glomerulus. These images show that dapagliflozin is filtered by glomeruli in the outer renal cortex and then binds to SGLT2 in the early proximal tubule. Scale bar, 100 μm. Parts (**b–d**) are adapted with permission of American Society of Nephrology from [32]; permission conveyed through Copyright Clearance Center, Inc. This figure is available as part of a downloadable slideset

Several studies have been carried out to confirm and explain the total inhibition of glucose reabsorption by phlorizin that is reported in humans [1]. For example, using microPET to monitor Me-4FDG excretion into the urinary bladder, we have shown that intravenous injection of phlorizin or the specific SGLT2 inhibitor dapagliflozin rapidly increases excretion of Me-4FDG into the urinary bladder (Fig. 5a) [32]. Dapagliflozin acts specifically on the kidneys, as shown by microPET imaging of mice injected with 4-[^18^F]fluoro-dapagliflozin (F-Dapa) [32]. F-Dapa binds specifically to the external surface of functional SGLT2 in the plasma membrane (with a binding constant of 4 nmol/l) [33] and is displaced by phlorizin and cold (non-radioactive) dapagliflozin. In rodents, the kidney is the only organ that shows significant specific F-Dapa binding (Fig. 5b). As SGLT2 inhibitors only bind to functional SGLT2 proteins in plasma membranes [31], this extends the immunohistochemical finding of the kidney-specific location of SGLT2 in rodents [23].

So, where in the kidney does F-Dapa bind? This has been determined by ex vivo autoradiography (Fig. 5c,d). The inhibitor binds to the outer cortex of the whole mouse kidney and (as shown at higher magnification) only within tubules surrounding the glomeruli. This pattern of binding is similar to the hybridisation of *Sglt2* (also known as *Slc5a2*) mRNA in rat kidneys and SGLT2 antibody binding in mouse, rat and human kidneys. We conclude that dapagliflozin is filtered from the plasma by the glomerulus and then binds to SGLT2 in the early proximal tubule, where it inhibits glucose absorption. Biochemical studies in dog kidney have shown that [^3^H]-phlorizin is filtered by the kidney, binds to the proximal tubule brush border membranes, and is displaced and excreted into the urine by excess cold (non-radioactive) phlorizin [34]. Unlike phlorizin, dapagliflozin and other SGLT2 inhibitors are not excreted into the urine, implying that they are reabsorbed further down the nephron and excreted in bile [32].

## Molecular mechanisms of glucose transport by SGLTs and GLUTs

**Fig. 6:**
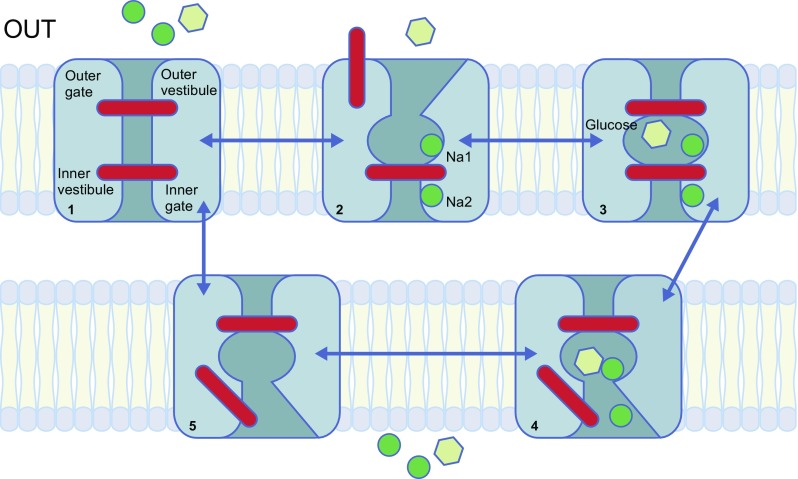
A mechanical model for sodium-coupled sugar transport. Sodium (green circle) binds first to the extracellular side (‘OUT’; state 1) to open the outer gate (state 2), permitting the sugar (glucose; yellow hexagon) to bind and be trapped in the bound site (state 3). The binding of both substrates induces a conformational change to an ‘inward facing’ conformation, resulting in the opening of the inner gate (state 4) and the release of Na^+^ and sugar into the cell interior. After the release of both substrates, the inner gate closes to form the inward facing ligand-free conformation (state 5). The cycle is completed by the change in conformation to the outward facing ligand-free (state 1). Phlorizin binds at the second point in the process (state 2). Adapted from [6], distributed under the terms of the CC BY 4.0 Attribution License (http://creativecommons.org/licenses/by/4.0/). This figure is available as part of a downloadable slideset

A mechanical model of sodium–glucose cotransport by SGLTs is shown in Fig. 6. The vast majority of information on SGLT-mediated sodium–glucose cotransport has come from biochemical and biophysical experiments on human SGLT1 in heterologous expression systems, with a more limited set of data from human SGLT2 experiments. In addition, solving the crystal structure of the bacterial homologue vSGLT and molecular dynamic simulations of these structures has provided further insights into the molecular mechanisms of SGLTs. We have also learnt from the fact that SGLTs belong to a structural family of transporters that have a common five-helix inverted repeat motif, the LeuT structural fold (see [6, 35]). Different cotransporters and exchangers have this common structure and so may have similar transport mechanisms. For cotransporters and exchangers in this family, the substrate binding sites are located in the middle of the protein, with external and internal gates isolating the substrate from the extracellular and intracellular solution on each side of the membrane (Fig. 6). Having determined the structure of GLUT transporters in the *SLC2* gene family [36], it is suggested that glucose transport through these proteins has a similar gated mechanism to the cotransporters, but with the difference that opening and closing of the external and internal gates is not controlled by sodium.

SGLT1 normally couples the inward transport of two sodium ions and one glucose molecule in each kinetic cycle. The direction of the transporter is completely reversible. The rate and direction of transport is simply a function of the extracellular and intracellular sodium and glucose concentrations and the polarity and magnitude of the membrane potential. Cotransport of sodium and glucose by SGLT1 and SGLT2 generates an electrical current and this provides a biophysical tool to measure the rate of cotransport. The sugar-activated (inward or outward) current is observed in the presence of sodium and is equivalent to the rate of sodium transport. In the case of SGLT1, capacitive currents are generated by voltage jumps in the presence and absence of sodium, in the absence of sugar. These currents are due to the movement of charged or polar amino acid residues of SGLT1 in the membrane electric field. They have provided a powerful biophysical means of measuring SGLT1 presteady state kinetics and the number of SGLT1 proteins in the plasma membrane. As yet, capacitive currents have not been recorded for human SGLT2.

In the normal forward transport cycle (Fig. 6), extracellular sodium first binds to the Na2 and Na1 sites. The Na2 site is conserved in SGLT2 and other members of the LeuT structural family: in SGLT1 the residues involved in coordinating Na^+^ are A76, I79, S289, S392 and S293. We have evidence that the Na1 site in human SGLT1 involves residues N78, H83, E102, Y290 and W291 [37]. Sodium binding increases the probability of opening the external gate so that external glucose (and phlorizin) can bind. The residues responsible for glucose binding are N78, H83, E102, K321, Q457, Y290 and W291, and these are conserved in human SGLT2. After binding glucose (or phlorizin) the outer gate closes to occlude the substrate from the external solution and then the inner gate opens to allow glucose and sodium to escape into the cytosol. Finally, the inner gate closes and the kinetic cycle continues to the initial starting position. We originally proposed an ordered internal dissociation of glucose and sodium, but recent experiments and molecular dynamic studies favour the simultaneous release of glucose and sodium [38]. One complete cycle takes about 20 ms.

Although kinetic studies of SGLT2 are not as advanced as those for SGLT1, the transport model is similar (Fig. 6). A major reason for the lack of data on SGLT2 is the low expression of this transporter in heterologous expression systems, such as *X. laevis* oocytes and cultured cells at 22°C. In preliminary kinetic studies on oocytes, the expression level of SGLT2 was less than 1% of that for SGLT1 [39]. On learning from Ana Pajor and Chari Smith that raising the temperature to 37°C dramatically increased SGLT2 activity in cultured cells [40], we were able to perform a robust comparison of the kinetics of SGLT1 and SGLT2 in HEK 293 cells [26, 33]. The major difference between SGLT2 and SGLT1 is that the sodium:glucose coupling ratio is 1:1 for SGLT2 vs 2:1 for SGLT1 [26]. Other differences include the higher affinity of phlorizin to SGLT2 vs SGLT1 (K_i_ 11 vs 140 nmol/l), the higher affinity of gliflozins, such as dapagliflozin to SGLT2 (K_i_ 4 vs 400 nmol/l), and a narrower sugar selectivity of SGLT2 (galactose is a poor substrate for SGLT2). Michael Coady and his colleagues recently elucidated the reason for low SGLT2 expression in oocytes with the discovery that co-expression of MAP17 was required [41, 42]. Apart from small differences in kinetic variables (likely owing to the difference in temperature of culture conditions), the kinetic properties of SGLT2 expressed in oocytes (cultured at 22°C) were identical to those in HEK 293 cells (cultured at 37°C). The role of MAP17 in SGLT2 expression in oocytes is not yet understood.

## Regulation of SGLTs

In HEK 293T cells, SGLT2 is regulated over the short term by stimulation of protein kinases, Protein kinase A (PKA) and Protein kinase C (PKC) [43]. This reversible stimulation occurs with a half-time of 10 min and is due to a change in the maximum rate of transport. Insulin mimics the effect of PKA and PKC activation, but this does not occur with deletion of the only phosphorylation site on SGLT2, Ser^624^Ala. PKC and PKA also regulate human SGLT1 in cultured cells, resulting in rapid changes in the trafficking of an intracellular pool of transporters to the cell membrane. In oocytes, biophysical and electron microscopic studies show that PKA- and PKC-induced increases in SGLT1 activity is due to an increase in the number of SGLT1 proteins in the cell membrane, at a rate of 1 × 10^7^ molecules per second [6, 44]. There was no effect of insulin on human SGLT1 trafficking.

## Structure of SGLTs

**Fig. 7:**
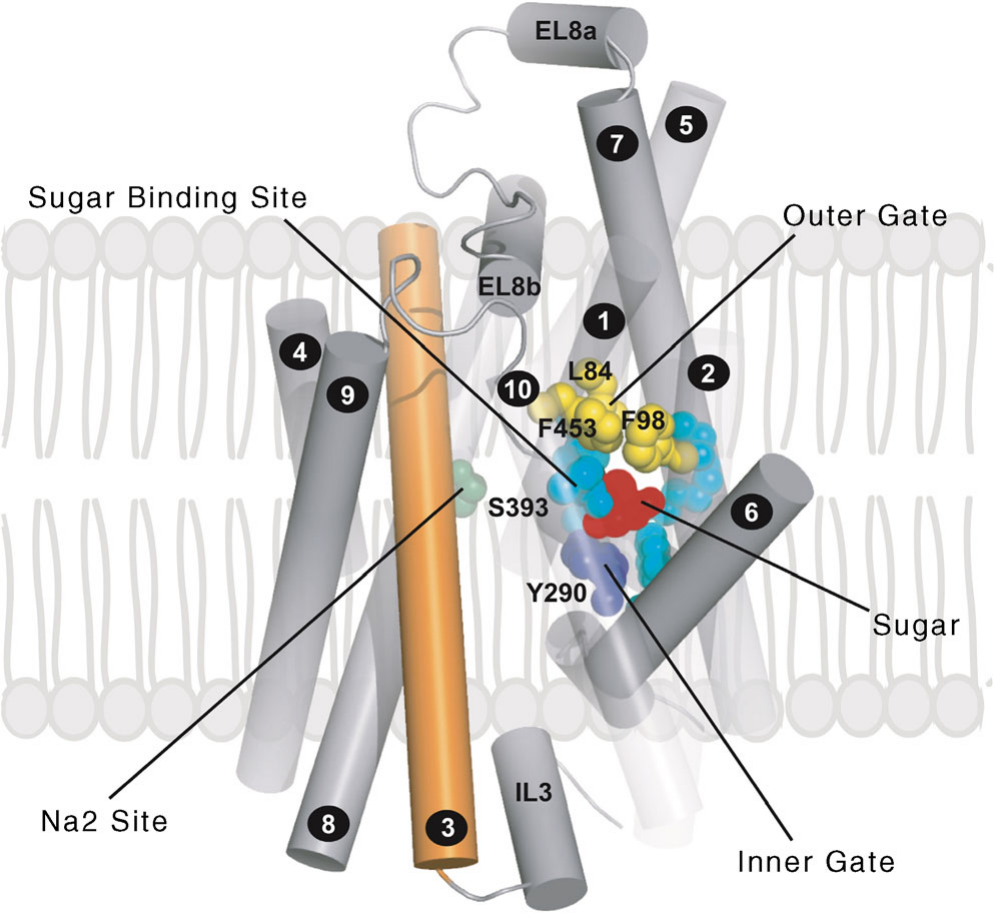
Homology model of the human SGLT2 based on the inward facing, occluded conformation of vSGLT (as described in [19]). Helices are represented as tubes. For clarity, helices −1 and 11–14 have been removed and helices 1, 2 and 10 are depicted as transparent. TM3 is coloured orange. Highlighted are the residues forming the glucose-binding site and the inner and outer gates EL8a and EL8b are helices in the external loop linking TM7 and TM8. This figure is available as part of a downloadable slideset

Following the work of Eric Turk in purifying the bacterial transporter, vSGLT [45], the x-ray structure was solved in two conformations [19, 20]. The structures have provided unique insights into the mechanism of sodium-coupled sugar transport, as incorporated into the transport model (Fig. 6). As aforementioned, the protein has 14 transmembrane helices with a core-inverted repeat of transmembrane helices 1–5 and 6–10, each containing a discontinuous helix (transmembrane helix [TM]1 and TM6). The substrate binding site is in the middle of the protein, adjacent to the discontinuous helices (N78, H83, E102, K321 Q457, Y290 and W291), and occluded from the external and internal solutions by external and internal gates. The relatively high sequence identities and similarities between vSGLT, SGLT1 and SGLT2 have made it possible to construct homology models of the human proteins (Fig. 7). In the sugar occluded state (Fig. 6, state 3) glucose is coordinated by N78, H83, E102, K321, Q457, Y290 and W291, and is excluded from contact with the external solution by hydrophobic residues (L84, F98 and F453) and the internal solution, in part, by Y290. A common Na2 sodium-binding site in the SGLT1 membrane and other proteins within the LeuT structural family is proposed at S393 (along with A76, I79, S389 and S392; not shown in Fig. 6). Mutations of each of the SGLT1 sugar and sodium coordination residues dramatically alter sugar binding, whereas mutations of the external gate residues do not [46].

Considerable progress has been made by using the structure of vSGLT to examine the molecular dynamics of SGLTs (interested readers are referred to the literature, e.g. [20, 38]). These and other biophysical studies of human SGLT1 provide insights into the conformational changes associated with sodium binding and isomerisation of the transporter between the outward and inward facing structures (e.g. see [37, 47]).

## Inhibitors of glucose reabsorption

**Fig. 8:**
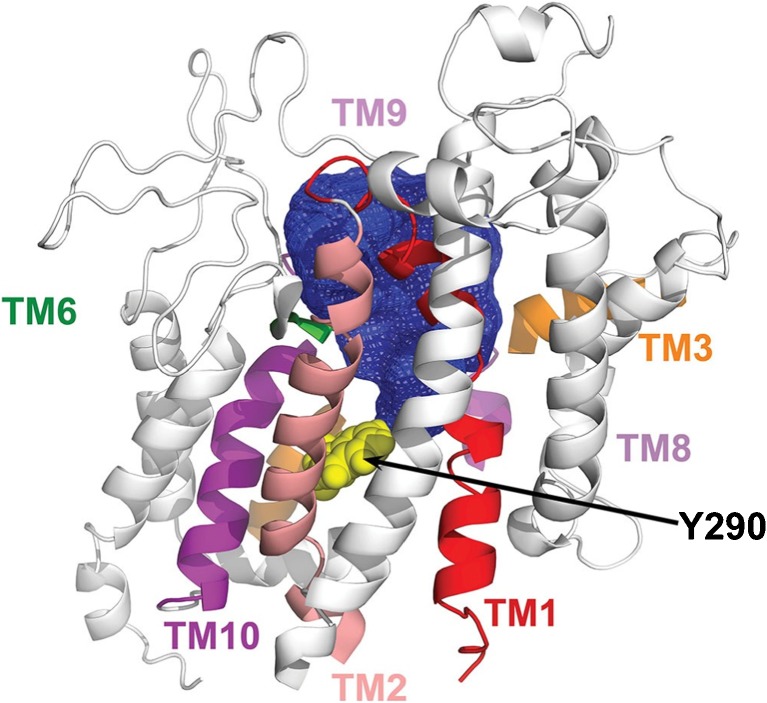
The external vestibule of human SGLT1 in the outward facing, sodium-bound conformation (Fig. 6, state 2). The vestibule was mapped using fluorescent reagents covalently bound to cysteine residues in the sugar-binding site, e.g. tetramethylrhodamine *(TAMRA*) bound to Y290C. The location of transmembrane helices (TM) of the structural model of SGLT1 are shown (some helices have been removed for clarity), along with the boundary of the 600 Å^3^ vestibule (blue area) bounded by the outer ends of TM1, TM2, TM3, TM6, TM9 and TM10. Reproduced from [47], distributed under the terms of the Creative Commons Attribution-NonCommerical-NoDerivatives International License 4.0 (CC BY-NC-ND; https://creativecommons.org/licenses/by-nc-nd/4.0/). This figure is available as part of a downloadable slideset

The lead compound for the development of SGLT drugs was phlorizin [48]. As indicated above, this plant glucoside is a non-transported, specific competitive inhibitor of SGLT2 and SGLT1, with a K_i_ of 11 nmol/l and 140 nmol/l, respectively. It preferentially binds to the external surface of the SGLTs in the presence of external sodium, blocking glucose transport (inward or outward). The aglycones of phlorizin (phloretin) and dapagliflozin are poor, non-competitive inhibitors of SGLTs, indicating that the glycosides bind to both the glucose-binding pocket and an adjacent lipophilic vestibule leading to the glucose-binding site (Fig. 8). There is remarkable selectivity in the binding of gliflozins to SGLTs; for example, dapagliflozin binds to SGLT2 with a much greater affinity than to SGLT1, and galacto-dapagliflozin has orders of magnitude lower affinity for SGLT1 than SGLT2 [33].

A mutational analysis of phlorizin binding to SGLT1 confirms that the sugar-binding site is important for both glucose and phlorizin binding, in that there was a linear relationship between glucose K_m_ and phlorizin K_i_ [46]. A major exception is that mutation of an outer gate residue, F101C, increased phlorizin K_i_ by 200-fold with no change in glucose K_m_. This indicates an interaction, possibly π–π bonding, between F101 and the phlorizin aglycone. Additional clues about the location of the phlorizin aglycone binding site comes from voltage clamp fluorometery of SGLT1, which identified that a 600 Å^3^ vestibule leads to the glucose-binding site in the sodium-bound open conformation (Fig. 6, state 2, and Fig. 8) [47]. This vestibule is lined with hydrophobic resides on TM1, TM2, TM6, TM9 and TM10 (Fig. 7). We anticipate that gliflozins bind to a similar site in both SGLT1 and SGLT2, and this is supported by molecular dynamic studies of inhibitor binding to SGLTs [49]. The success of SGLT2 drugs is, in large part, due to their high affinity for SGLT2 and the limited expression of this transporter in the kidney cortex (see above).

The development of GLUT2 drugs to treat diabetes has not been practical owing to the close structural and functional similarity between GLUT family members and the vital functions of GLUTs throughout the body [36].

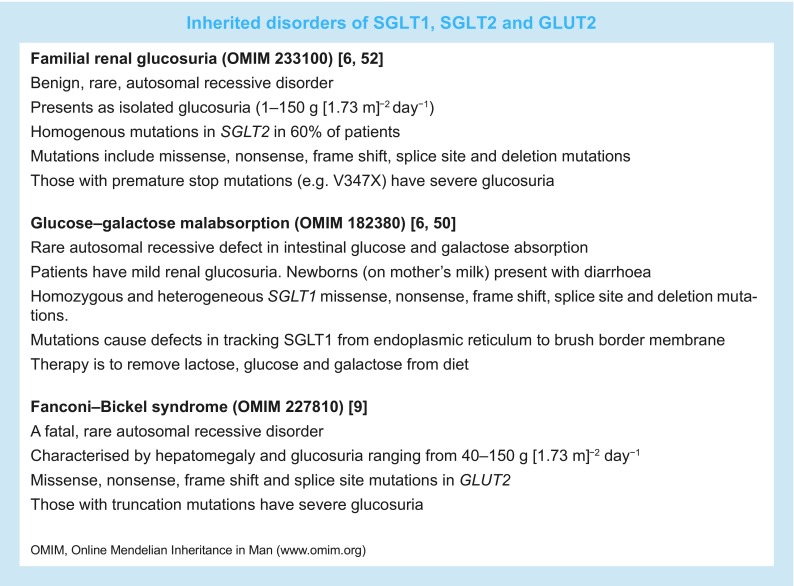



## Inherited disorders of renal glucose transporters

As previously mentioned, there are three known rare, autosomal recessive disorders of SGLTs and GLUT2, glucose–galactose malabsorption (OMIM 182380), familial renal glucosuria (OMIM 233100) and Fanconi–Bickel syndrome (OMIM 227810), all of which result in mild to excessive glucosuria (1–150 g [1.73 m]^−2^ day^−1^ [see textbox below]). In glucose–galactose malabsorption, mutations in *SGLT1* cause a defect in intestinal glucose (and galactose) absorption, resulting in mild glucosuria (see [6, 50]). These mutations cause malabsorption due to mistrafficking of SGLT1 to the brush border membrane. In contrast, mutations in *SGLT2* cause renal glucosuria (from 1 g [1.73 m]^−2^ day^−1^ to 150 g [1.73 m]^−2^ day^−1^) without defects in intestinal absorption. Only in cases of homogeneous truncation mutations in *SGLT2* is it reasonable to expect severe glucosuria, but the reserve capacity of SGLT1 should be taken into account. Unlike glucose–galactose malabsorption, there are no comprehensive studies of the transport properties of SGLT2 mutants, largely due to the low expression of SGLT2 in heterologous expression systems (as detailed above). This, and the incomplete clinical study of individuals with familial renal glucosuria, has led to confusion about the inheritance of this disease. However, we expect familial renal glucosuria to be a simple autosomal recessive disease.

As with SGLT2, truncation mutations of *GLUT2* are expected to cause complete excretion of the filtered glucose load if GLUT2 is the major glucose transporter on the proximal tubule basolateral membrane. However, the phenotype of Fanconi–Bickel syndrome is complex owing to the importance of GLUT2 in the liver and other organs.

Overall, these genetic disorders, caused by mutations in *SGLT1*, *SGLT2* and *GLUT2*, support animal studies and confirm the importance of the three affected proteins in reabsorbing the filtered glucose load in healthy humans.

## Summary

Thirty years ago, the cloning of human SGLT1, SGLT2 and GLUT2 was a major breakthrough in the identification of transporters involved in renal glucose absorption. Subsequent advances in molecular biology permitted the development of genetic mouse models lacking SGLT1, SGLT2 and GLUT2, furthering our understanding of the role of these transporters in the proximal tubule. Based on the assumption that SGLT2 was responsible for the bulk of glucose reabsorption, the pharmaceutical industry in Japan, USA and Europe invented SGLT2 inhibitors to treat type 2 diabetes. Although these drugs do lower blood glucose levels in patients with diabetes without causing hypoglycaemia, they are only capable of a 50% inhibition of glucose reabsorption and are, thus, not so effective as a monotherapy. In retrospect, given the huge reserve capacity of SGLT1 in the late proximal tubule, one can argue that dual SGLT2 and SGLT1 drugs may be more effective in controlling renal glucose excretion.

## Electronic supplementary material

ESM Downloadable slideset (PPTX 5353 kb)

## Abbreviations

2-FDG: 2-Deoxy-2-fluoro-[^18^F]-d-glucose
F-Dapa: 4-[^18^F]fluoro-dapagliflozin
HEK 293: Human embryonic kidney cells
K_i_: Inhibitor constant
K_m_: Apparent affinity constant
Me-4FDG: Methyl-4-fluoro-[^18^F]-4-deoxy-d-glucopyranoside
OMIM: Online Mendelian Inheritance in Man
PET: Positron emission tomography
PKA: Protein kinase A
PKC: Protein kinase C
SGLT: Sodium−glucose cotransporter
TM: Transmembrane helix
