# Supplementary material for: Physiology of renal glucose handling via SGLT1, SGLT2 and GLUT2
Source: Diabetologia. 2018 Aug 22;61(10):2087–97. doi: 10.1007/s00125-018-4656-5 (PMC6133168; doi:10.1007/s00125-018-4656-5)
Supplement: Supplementary file 1 — (PPTX 5353 kb) [file 125_2018_4656_MOESM1_ESM.pptx]

## Slide 1
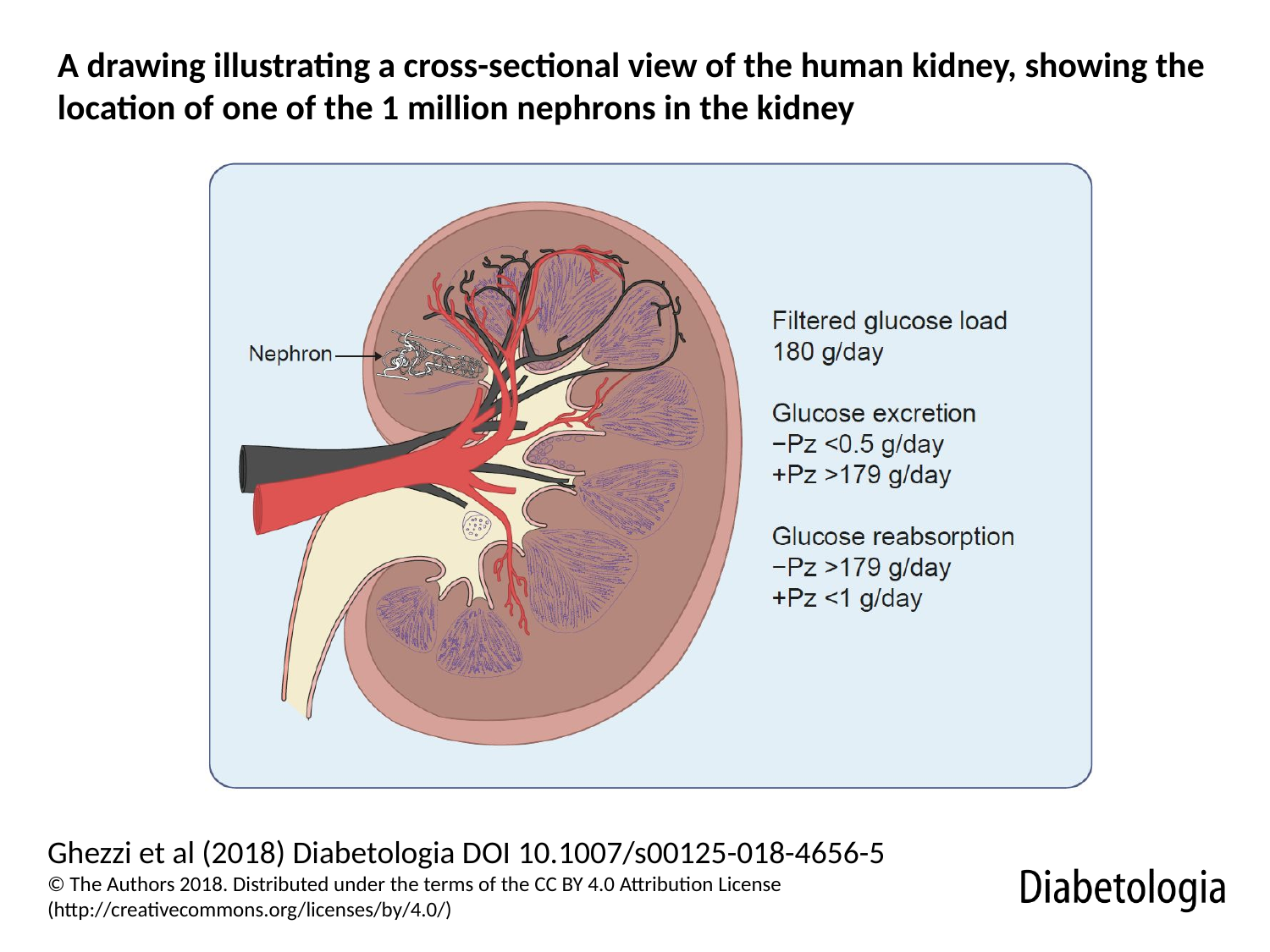

A drawing illustrating a cross-sectional view of the human kidney, showing the location of one of the 1 million nephrons in the kidney
Ghezzi et al (2018) Diabetologia DOI 10.1007/s00125-018-4656-5
© The Authors 2018. Distributed under the terms of the CC BY 4.0 Attribution License (http://creativecommons.org/licenses/by/4.0/)

## Slide 2
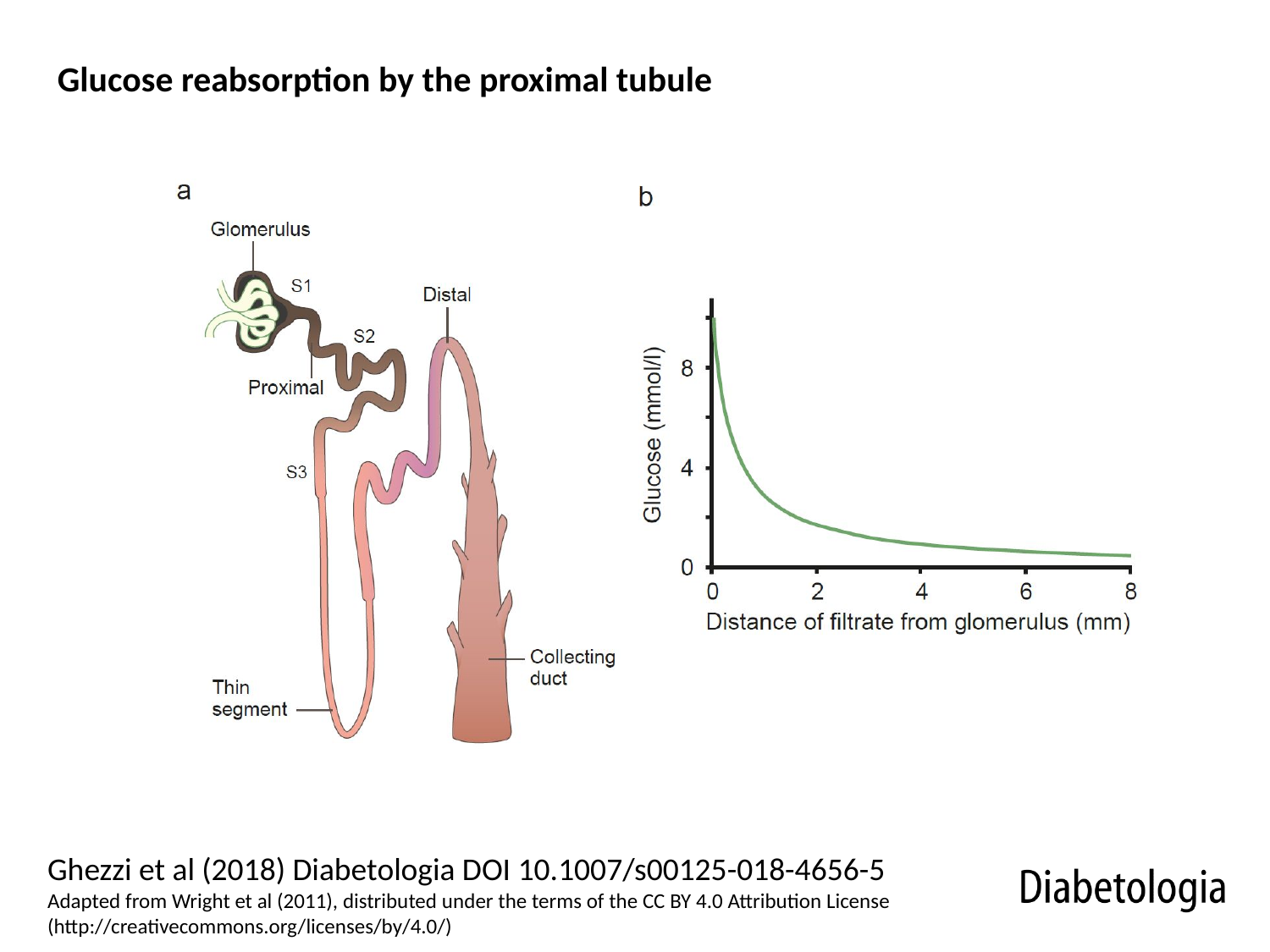

Glucose reabsorption by the proximal tubule
Ghezzi et al (2018) Diabetologia DOI 10.1007/s00125-018-4656-5
Adapted from Wright et al (2011), distributed under the terms of the CC BY 4.0 Attribution License (http://creativecommons.org/licenses/by/4.0/)

## Slide 3
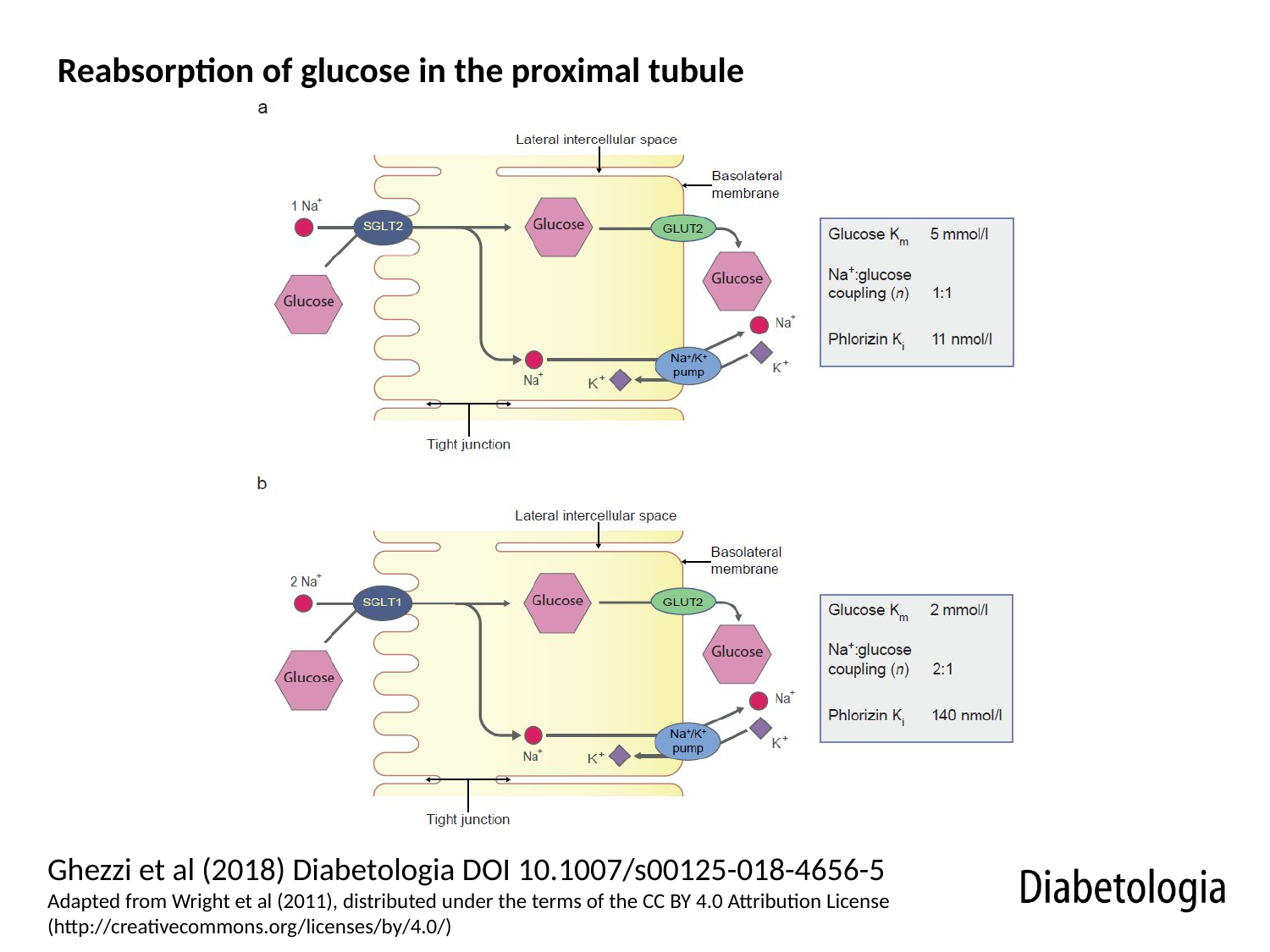

Reabsorption of glucose in the proximal tubule
Ghezzi et al (2018) Diabetologia DOI 10.1007/s00125-018-4656-5
Adapted from Wright et al (2011), distributed under the terms of the CC BY 4.0 Attribution License (http://creativecommons.org/licenses/by/4.0/)

## Slide 4
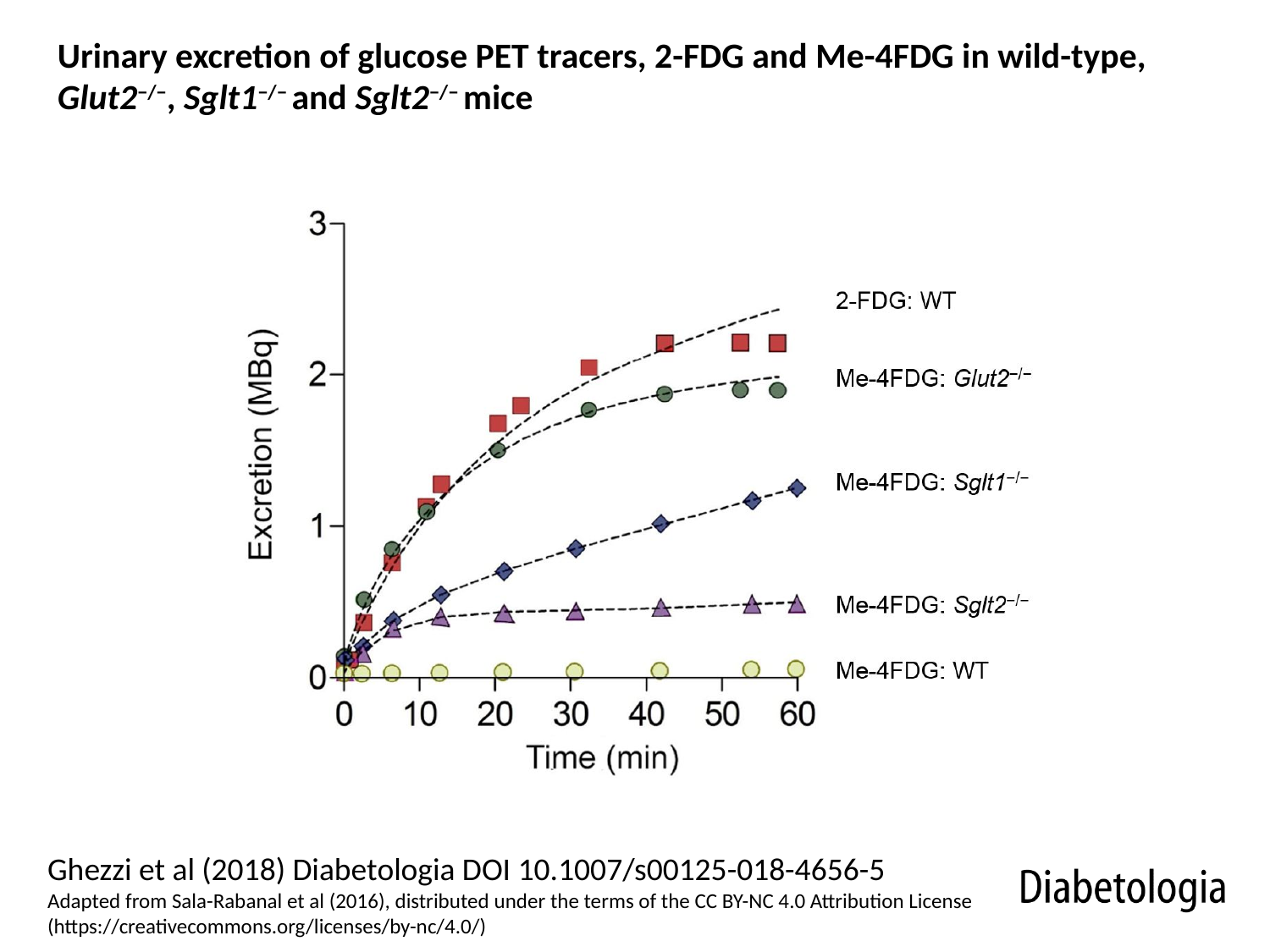

Urinary excretion of glucose PET tracers, 2-FDG and Me-4FDG in wild-type, Glut2−/−, Sglt1−/− and Sglt2−/− mice
Ghezzi et al (2018) Diabetologia DOI 10.1007/s00125-018-4656-5
Adapted from Sala-Rabanal et al (2016), distributed under the terms of the CC BY-NC 4.0 Attribution License (https://creativecommons.org/licenses/by-nc/4.0/)

## Slide 5
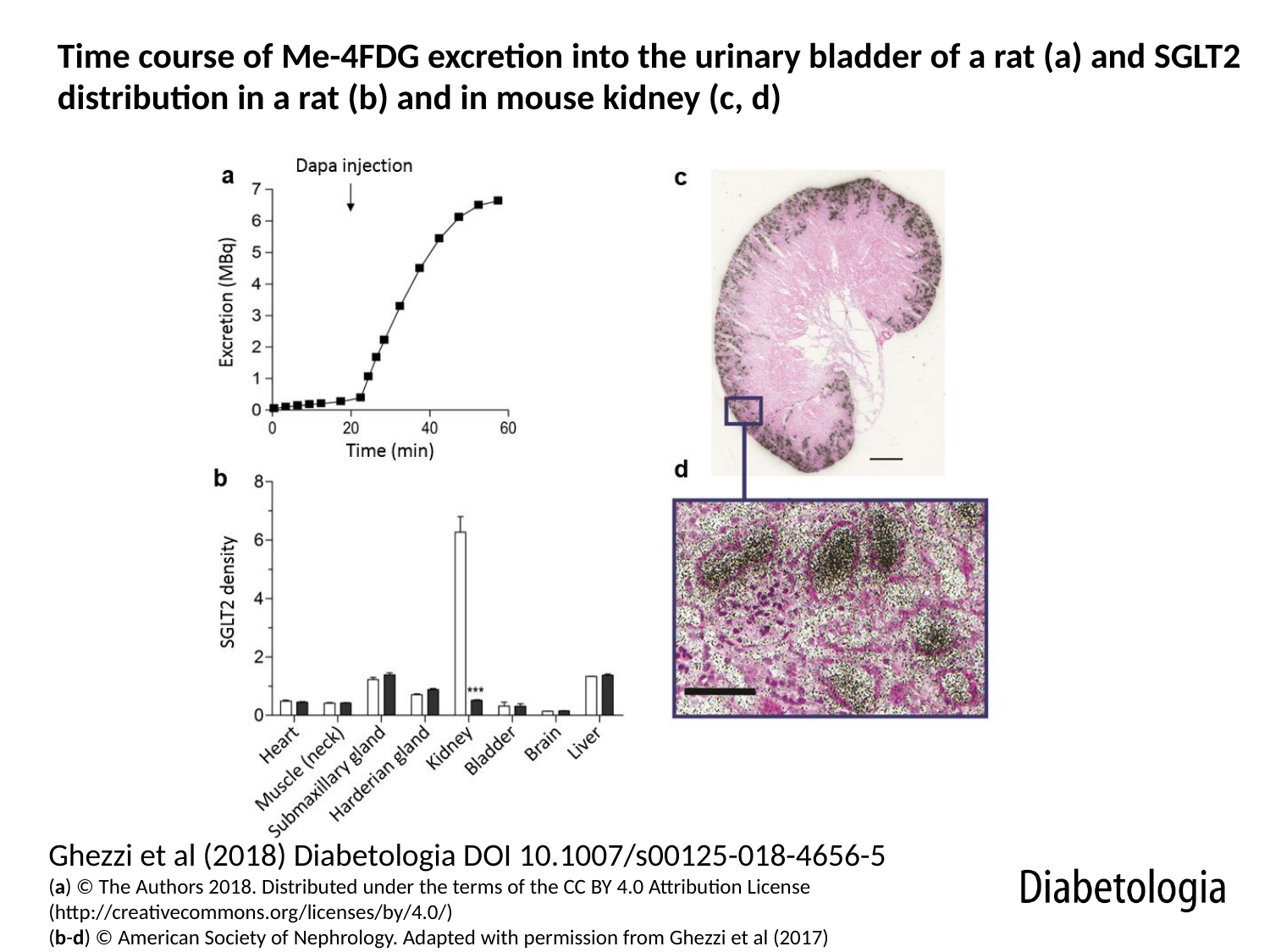

Time course of Me-4FDG excretion into the urinary bladder of a rat (a) and SGLT2 distribution in a rat (b) and in mouse kidney (c, d)
Ghezzi et al (2018) Diabetologia DOI 10.1007/s00125-018-4656-5
(a) © The Authors 2018. Distributed under the terms of the CC BY 4.0 Attribution License (http://creativecommons.org/licenses/by/4.0/)
(b-d) © American Society of Nephrology. Adapted with permission from Ghezzi et al (2017)

## Slide 6
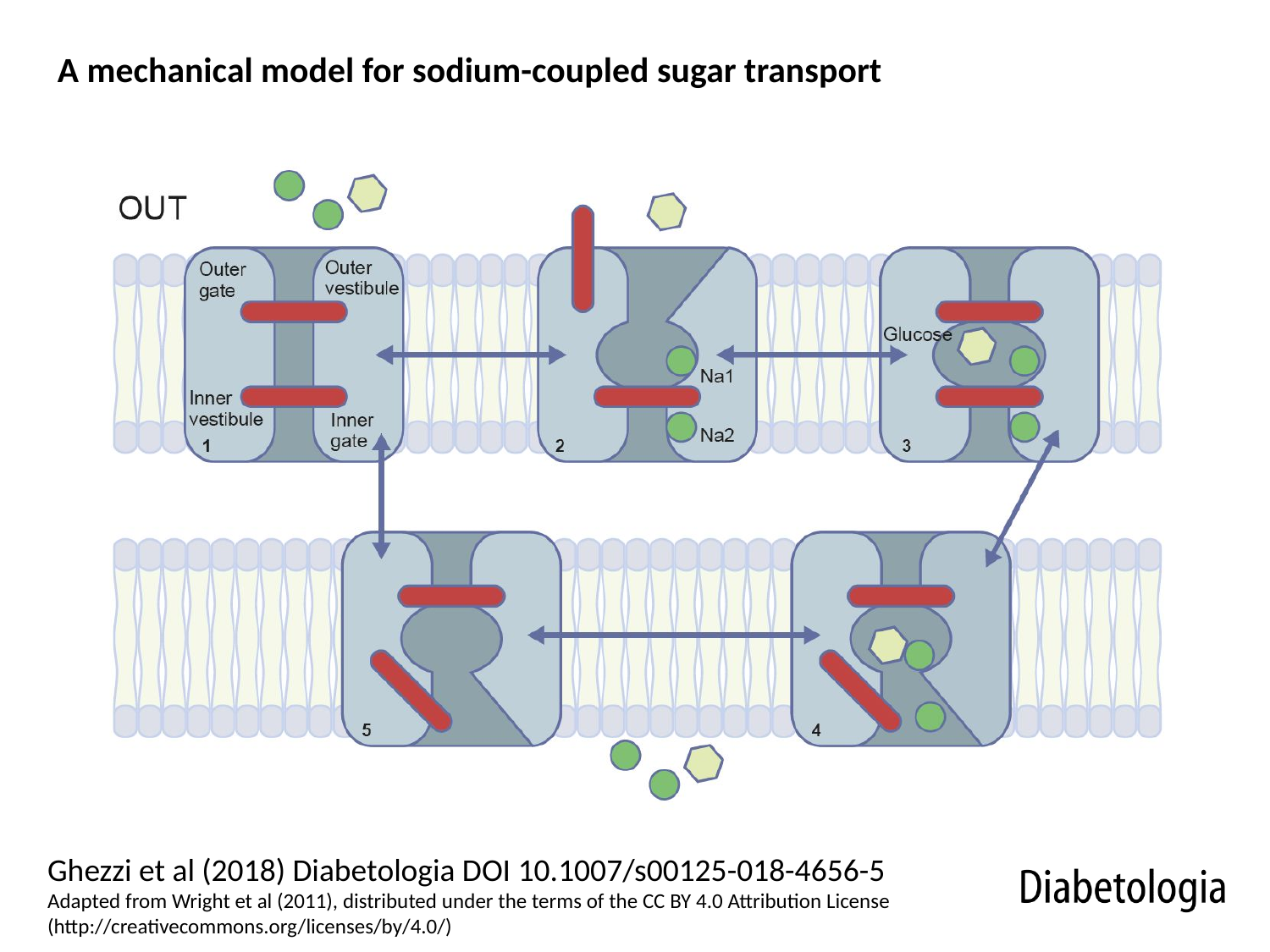

A mechanical model for sodium-coupled sugar transport
Ghezzi et al (2018) Diabetologia DOI 10.1007/s00125-018-4656-5
Adapted from Wright et al (2011), distributed under the terms of the CC BY 4.0 Attribution License (http://creativecommons.org/licenses/by/4.0/)

## Slide 7
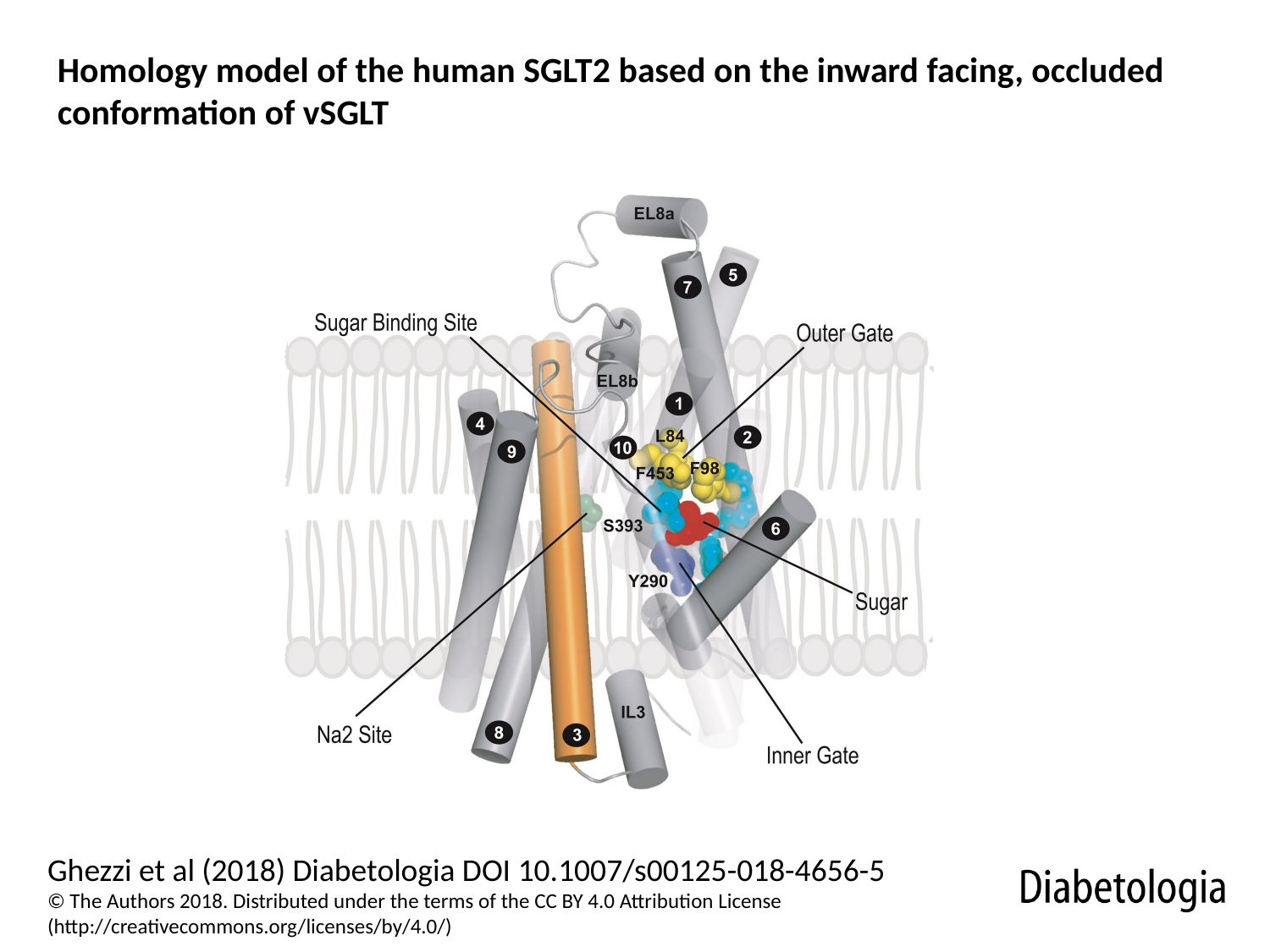

Homology model of the human SGLT2 based on the inward facing, occluded conformation of vSGLT
Ghezzi et al (2018) Diabetologia DOI 10.1007/s00125-018-4656-5
© The Authors 2018. Distributed under the terms of the CC BY 4.0 Attribution License (http://creativecommons.org/licenses/by/4.0/)

## Slide 8
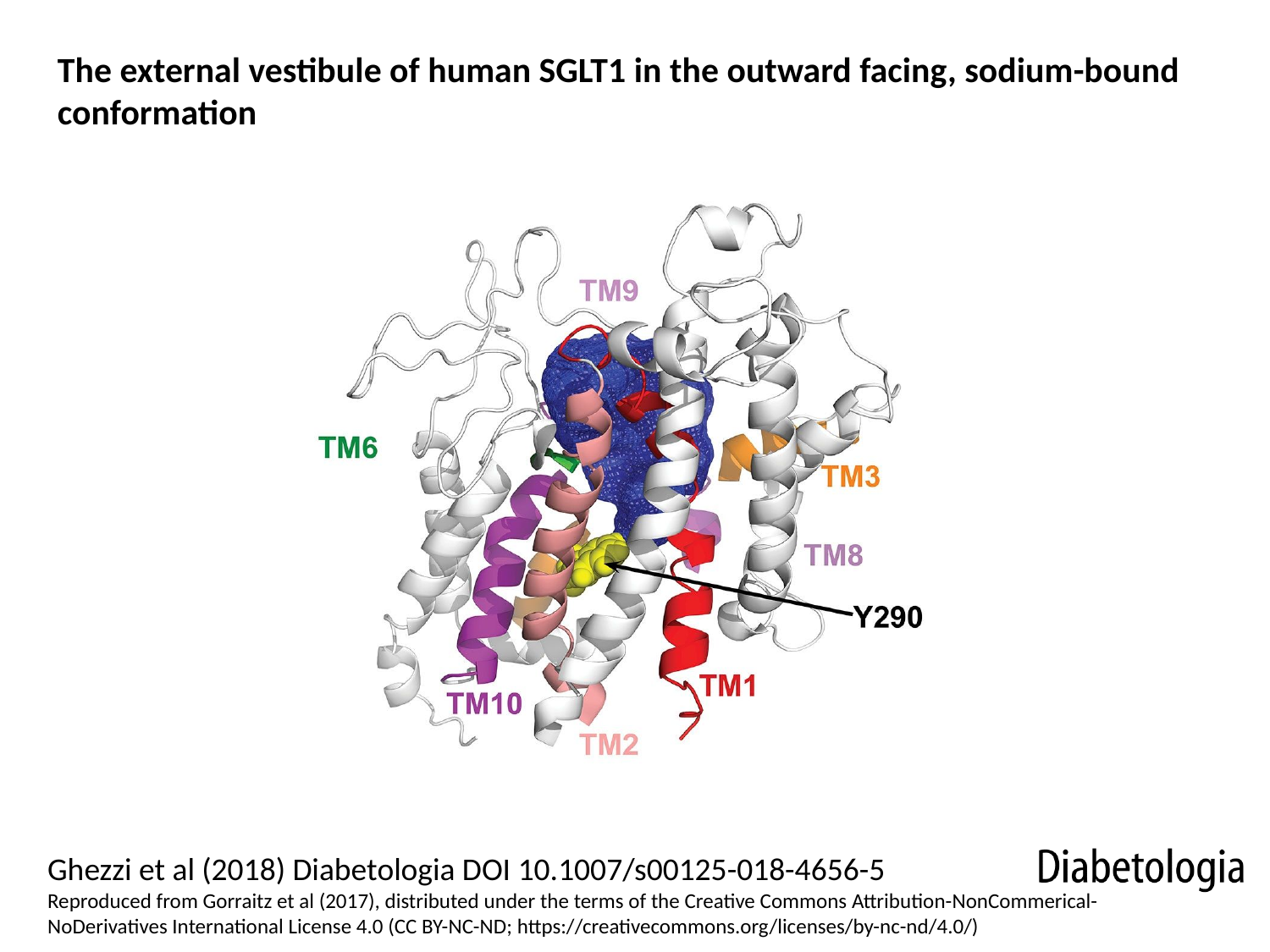

The external vestibule of human SGLT1 in the outward facing, sodium-bound conformation
Ghezzi et al (2018) Diabetologia DOI 10.1007/s00125-018-4656-5
Reproduced from Gorraitz et al (2017), distributed under the terms of the Creative Commons Attribution-NonCommerical-NoDerivatives International License 4.0 (CC BY-NC-ND; https://creativecommons.org/licenses/by-nc-nd/4.0/)
